# Timing skills and expertise: discrete and continuous timed movements among musicians and athletes

**DOI:** 10.3389/fpsyg.2014.01482

**Published:** 2014-12-23

**Authors:** Thenille Braun Janzen, William Forde Thompson, Paolo Ammirante, Ronald Ranvaud

**Affiliations:** ^1^Department of Psychology, Macquarie UniversitySydney, NSW, Australia; ^2^Department of Neuroscience and Behavior, Institute of Psychology, University of São PauloSão Paulo, Brazil; ^3^Department of Psychology, Ryerson UniversityToronto, ON, Canada

**Keywords:** emergent timing, event timing, expertise, training, music, sports

## Abstract

**Introduction:** Movement-based expertise relies on precise timing of movements and the capacity to predict the timing of events. Music performance involves discrete rhythmic actions that adhere to regular cycles of timed events, whereas many sports involve continuous movements that are not timed in a cyclical manner. It has been proposed that the precision of discrete movements relies on event timing (clock mechanism), whereas continuous movements are controlled by emergent timing. We examined whether movement-based expertise influences the timing mode adopted to maintain precise rhythmic actions.

**Materials and Method:** Timing precision was evaluated in musicians, athletes and control participants. Discrete and continuous movements were assessed using finger-tapping and circle-drawing tasks, respectively, based on the synchronization-continuation paradigm. In Experiment 1, no auditory feedback was provided in the continuation phase of the trials, whereas in Experiment 2 every action triggered a feedback tone.

**Results:** Analysis of precision in the continuation phase indicated that athletes performed significantly better than musicians and controls in the circle-drawing task, whereas musicians were more precise than controls in the finger tapping task. Interestingly, musicians were also more precise than controls in the circle-drawing task. Results also showed that the timing mode adopted was dependent on expertise and the presence of auditory feedback.

**Discussion:** Results showed that movement-based expertise is associated with enhanced timing, but these effects depend on the nature of the training. Expertise was found to influence the timing strategy adopted to maintain precise rhythmic movements, suggesting that event and emergent timing mechanisms are not strictly tied to specific tasks, but can both be adopted to achieve precise timing.

## Introduction

Experts such as musicians and athletes rely on precise timing of bodily movements. However, whereas musicians are especially skilled at discrete rhythmic actions that adhere to regular cycles of timed events (meter and pulse) (Repp and Doggett, 2007; Baer et al., 2013; Albrecht et al., 2014), athletic sports often involve fluid and continuous movements that are not timed in a cyclical manner (Sternad et al., 2000; Jaitner et al., 2001; Jantzen et al., 2008; Balague et al., 2013). Research suggests that the timing of discrete movements (i.e., those preceded and followed by a period without motion) and continuous movements depend on different strategies or processes (Robertson et al., 1999; Zelaznik et al., 2002; Huys et al., 2008; Zelaznik and Rosenbaum, 2010; Studenka et al., 2012). Specifically, the timing of discrete movements is thought to involve a clock-like mechanism that incorporates an explicit representation of the time interval delineated by each discrete movement. In contrast, activities that involve smooth and continuous rhythmic movements are thought to be based on emergent timing, whereby timing regularity emerges in the absence of a representation of time interval from the control of parameters such as movement trajectory and velocity.

The hypothesis that event and emergent timing are distinct and dissociable systems is supported by a substantial body of evidence. Behavioral studies have shown that temporal variability in finger tapping is usually uncorrelated with variability in continuous circle drawing (Robertson et al., 1999; Zelaznik et al., 2005), and that event-timed movements, such as tapping, are significantly more precise and adjust faster to timing perturbations than continuous movements such as circle drawing (Elliot et al., 2009; Repp and Steinman, 2010; Studenka and Zelaznik, 2011). There is also neurological (Ivry et al., 2002; Spencer et al., 2003, 2005) and neuroimaging (Schaal et al., 2004; Spencer et al., 2007) evidence that event and emergent timing processes recruit different brain areas.

However, recent results have raised doubts that discrete and continuous movements always engage event and emergent timing mechanisms, respectively (Jantzen et al., 2002, 2004; Repp and Steinman, 2010; Studenka et al., 2012; Studenka, 2014). For example, evidence suggests that the presence of perceptual events marking the completion of time intervals can induce event timing even for tasks performed with continuous movements (Zelaznik and Rosenbaum, 2010; Studenka et al., 2012). Computational simulations and behavioral studies also suggest that task tempo and movement speed constraints (Huys et al., 2008; Zelaznik and Rosenbaum, 2010), as well as task order and practice (Jantzen et al., 2002, 2004), are important influences on the timing mechanism adopted for a certain task. Based on the suggestion that the timing mechanisms recruited to perform rhythmic movements are significantly influenced by several factors, the present investigation tested whether two distinct forms of expertise and training (music and sport) differentially influence the strategy that is engaged to perform movement-based timing tasks.

Practice is generally regarded in the motor learning literature as one of the most essential predictors of motor skill acquisition (Schmidt and Lee, 1988; Smith, 2003; Tenenbaum and Eklund, 2007; but see Mosing et al., 2014) and researchers have suggested that the amount of deliberate practice is directly associated with the level of expertise acquired by athletes and musicians (Ericsson et al., 1993; Ericsson, 1996; Howe et al., 1998). It is well known that highly trained musicians are exceptionally precise in discrete-timing tasks, such as finger tapping with an auditory metronome (Repp, 2005, 2010; Repp and Doggett, 2007; Baer et al., 2013). Musicians tend to show smaller asynchronies and lower tapping variability when tapping to a metronome compared with non-musician counterparts (Aschersleben, 2002; Repp, 2010). Musical expertise also seems to enhance the internal representation of time as suggested by perceptual studies showing that training can improve interval discrimination and perceptual sensitivity to timing perturbations (Buonomano and Karmakar, 2002; Ivry and Schlerf, 2008; Repp, 2010). Research also demonstrates that musicianship specifically interacts with tasks associated with discrete movements, and not continuous movements (Baer et al., 2013), which is consistent with the view that emergent and event timing are distinct mechanisms (Zelaznik et al., 2000, 2005) and suggests that music expertise is predominantly an event-based skill (Repp, 2005; Baer et al., 2013).

On the other hand, we know very little about how expertise and training might influence the operation of emergent timing mechanisms, and whether the effect of training in one movement-based expertise might transfer to other timing skills. The timing of continuous rhythmic movements, such as leg movement during cycling, walking and running, or arm movements during swimming or rowing, is thought to rely on emergent timing mechanisms (Kelso et al., 1981; Sternad et al., 2000; Jaitner et al., 2001; Jantzen et al., 2008; Elliot et al., 2009; Balague et al., 2013). This class of rhythmic movements is typically observed in sport activities such as rowing, swimming, running, and cycling, and could therefore be used as a model to study the effect of training in the production of precise continuous rhythmic movements. The purpose of the present study was to compare the ability of movement-based experts from different domains to engage in discrete and continuous movement tasks. Based on the hypothesis that musical performance involves predominantly discrete rhythmic actions that rely on event timing, and that athletic sports generally recruit fluid and continuous rhythmic movements based on emergent timing, we examined whether movement-based expertise is associated with specific or general timing skills. If the event and emergent timing processes are dependent on the nature of expertise and training, then athletes should be more precise in a timing task that involves continuous movements whereas musicians should be more precise in a timing task that involves discrete movements. In contrast, if musicians and athletes do not differ in their performance in both tasks, then this would suggest that timed movements are accomplished similarly in these two groups of movement-based experts and, therefore, that event and emergent timing mechanisms are not strictly tied to specific tasks, but may both be adopted to achieve precise timing.

Experiment 1 compared the performance of elite athletes, highly trained musicians, and controls on finger-tapping and circle-drawing tasks. The variability of inter-response intervals was measured in a synchronization-continuation paradigm. Participants were instructed to synchronize their movements with a metronome and continue the action at the same rate established by the metronome even when the pacing signal stopped (continuation phase). In Experiment 2, auditory feedback was presented in the continuation phase in order to assess the effect of the presence of salient perceptual events on the timing mechanism adopted to complete the tasks. Based on past research (Zelaznik and Rosenbaum, 2010; Studenka et al., 2012; Baer et al., 2013), we predicted that the presence of auditory feedback would induce an event-timing strategy in the continuous movement task, regardless of the expertise of the participants.

## Experiment 1

### Materials and methods

#### Participants

Fifteen athletes were recruited through the Macquarie University Elite Athlete Scholarship Program. Athletes (8 females, 7 males) were on average 21.31 years old (*SD* = 2.33, range 18–26 years) and had been involved in athletic training for an average of 7.31 years (SD = 3.45). All athletes involved in the project were actively engaged in training and competing at State and/or National level in athletic sports, such as swimming, rowing, martial arts, rugby and others. None of the athletes had completed more than 2 years of musical training or were involved in any musical activities. Musicians (*n* = 13, 4 females) were recruited through the Departments of Music and Psychology at Macquarie University and local conservatories and universities. The average age of musicians was 21.38 years (*SD* = 3.20, range 18–28 years) and all participants had been involved in formal music training for at least 10 consecutive years (*M* = 10.85, *SD* = 2.38). Musicians played a range of instruments, including piano, guitar, and violin. Control participants (*n* = 17, 10 females) were on average 21.76 years old (*SD* = 3.31, range 18–31 years). None of the participants in the control group reported any formal athletic or music training. Groups did not differ significantly in mean age, *F*_(2, 42)_ = 0.07, *p* = 0.93. All participants reported that they were right-handed and had no hearing or motor impairment. Psychology undergraduate students were compensated with course credit, and all other participants received financial compensation for their participation. All participants provided informed consent and were debriefed about the goals of the experiment.

#### Materials, stimuli, and procedure

Stimulus presentation and data collection were done using a Macbook Pro 9.2 laptop running custom software written in Python and tasks were completed using an Apple single-button mouse. The task widely used to induce event timing is finger tapping, whereas circle drawing is thought to typify emergent timing (Repp and Steinman, 2010). The paradigm adopted for both tasks was synchronization-continuation (Stevens, 1886). For each trial, participants first synchronized their movements (circle drawing or finger tapping) with an isochronous metronome click for 18 clicks. The signal tones were 40 ms square waves clicks of 480 Hz presented at 74 dB. After the synchronization phase, the metronome stopped and participants were instructed to continue to produce 36 more movements at the tempo set by the metronome. Within each trial, one of two metronome tempi was used: slow (800 ms IOI) or fast (600 ms IOI).

In the finger-tapping task, participants repeatedly tapped on the mouse with their right index finger at the tempo set by the metronome pacing signals and continued to tap at the same rate when the signal was removed. Participants heard the pacing signals through Sennheiser HD 515 headphones with noise canceling and reduction, which prevented participants from hearing any sound produced by the finger tap. No auditory feedback was provided.

In the circle-drawing task, participants repeatedly moved the computer mouse with the right hand in a circle in time with the metronome and in a clockwise direction, and continued this motion in the absence of the external timing cue. Participants traced an unfilled circle template of 5 cm in diameter displayed on the screen with the mouse cursor, and were instructed to synchronize every time the path of the cursor crossed an intersection at 270° of the circle with the metronome. Participants were told that timing precision was more relevant than drawing accuracy, and they were free to draw a circle at their preferred size.

Participants had 5 practice trials at 600 ms IOI before each experimental block. Trials were blocked by task, with tapping performed before circle drawing (Zelaznik and Rosenbaum, 2010; Studenka et al., 2012). For each task, trials were blocked by tempo, with the order of the two tempo conditions and the 10 trials within each tempo condition randomized independently for each participant. Participants were permitted to take breaks in between trials at any time. The experiment took approximately 50 min.

#### Data analysis

Only responses in the continuation phase were analyzed as the synchronization phase was used only to establish the pacing. In order to allow for acceleration commonly observed in the transition from the synchronization to continuation phase (Flach, 2005), only the final 30 movements were analyzed. For the finger-tapping task, inter-response interval (IRI) was defined as the elapsed time between sequential taps (in milliseconds) and for the circle-drawing task, IRI was defined as the elapsed time between successive passes through the intersection. Outlier IRIs were identified as those 60% longer or shorter than the target IRI for a given trial (4% of all IRIs analyzed in Experiment 1; 2% in Experiment 2), and were deleted.

Several timing measures were used. First, mean IRI within a trial served as a measure of timing accuracy. Second, to measure timing precision we analyzed participants coefficient of variation (CV), which was defined as the standard deviation of IRIs within a trial divided by its mean IRI (SD/Mean). Lower CV scores indicate greater precision. CV can be considered a measure of total IRI variability, including slow drift in IRI over the course of a trial, timing error, and motor implementation error. Third, dependencies between successive IRIs in each trial were measured using lag-one autocorrelation. Data were first linearly detrended to remove the impact of slower drift over the course of a trial on dependencies between successive IRIs. In general, discrete-timing tasks are associated with negative lag-one autocorrelation. This has been proposed to arise from random delays in motor implementation (Wing and Kristofferson, 1973) that occur independently of a central clock mechanism. One such delay should both lengthen the IRI that it completes and shorten the one that it initiates; the accumulation of these delays over the course of a trial should be reflected in negative lag-one autocorrelation. Continuous-timing tasks, on the other hand, which are thought not to involve a central clock mechanism, have been shown to result in non-negative lag-one autocorrelation (Zelaznik and Rosenbaum, 2010; Baer et al., 2013). Thus, lag-one autocorrelation can serve as an index of event and emergent timing strategies. CV and lag-one autocorrelation values were averaged by task and tempo for each participant.

Finally, we sought to estimate clock and motor contributions to timing variance (Wing and Kristofferson, 1973) using slope analysis (Ivry and Hazeltine, 1995). Slope analysis takes advantage of the well-established finding that timing variance increases linearly as a function of squared target duration. Under the assumption that motor production is invariant across target durations, a positive slope (i.e., an increase in variance with target duration) is thought to be influenced entirely by duration-dependent variability (Studenka and Zelaznik, 2008). The intercept of this regression line, on the other hand, is thought to be duration-independent, i.e., reflecting variability in the motor aspect of the task (Studenka and Zelaznik, 2008). Different event-like tasks have been shown to exhibit equal slope values (Ivry and Hazeltine, 1995; Green et al., 1999), suggesting a common underlying central clock mechanism. On the other hand, (emergent) circle-drawing and (event) finger-tapping tasks have been shown to exhibit significantly different slopes (Robertson et al., 1999), suggesting different timing mechanisms. Individual differences in slope are also observed within tasks (Spencer et al., 2005; Baer et al., 2013), with lower slope values indicating less duration-dependent variability. In the current study, for each participant and for each task, slope and intercept values were obtained from a linear regression of detrended variance (averaged across trials) against squared target durations (600 and 800 ms^2^).

### Results

Preliminary analysis of mean IRI during the continuation phase revealed that participants were accurate in maintaining the target tempi [fast tempo (600 ms IOI): *M* = 606; *SD* = 35; slow tempo (800 ms IOI): *M* = 818; *SD* = 55]. There were no significant differences between groups or group interactions.

Coefficient of variation (CV) scores were entered into a mixed design ANOVA with Task (circle drawing, finger tapping) and Tempo (fast, slow) as within-subject factors and Group (athletes, musicians, controls) as between-subject factors. There was a significant main effect of Task, *F*_(1, 42)_ = 251.01, *p* < 0.001, demonstrating that participants were more precise in the finger-tapping task (*M* = 0.07) than the circle-drawing task (*M* = 0.23). It was also verified that there was no statistical difference in CV between the fast and slow tempi conditions, *F*_(1, 42)_ = 1.16, *p* = 0.28, and no significant interaction between Task and Tempo, *F*_(1, 42)_ = 2.25, *p* = 0.14.

**Figure 1:**
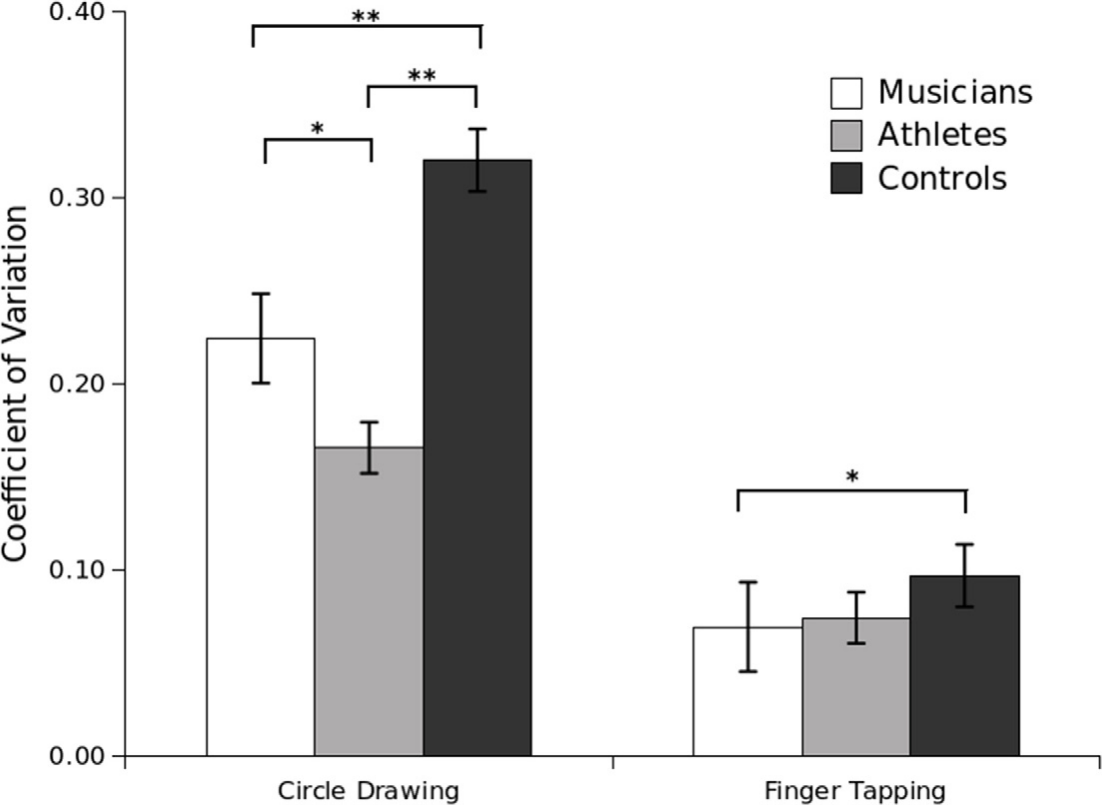
**Coefficient of Variation (CV) for the circle-drawing and finger-tapping tasks per group in Experiment 1**. Standard error bars are shown. Significant pairwise differences are marked with an asterisk (^*^p < 0.05; ^**^p < 0.001).

Between-subjects analysis revealed a significant main effect of Group, *F*_(2, 42)_ = 18.42, *p* < 0.001, and a significant interaction between Group and Task, *F*_(2, 42)_ = 16.48, *p* < 0.001. Independent sample *t*-tests revealed that on the circle-drawing task athletes were significantly more precise than musicians, *t*_(26)_ = 2.19, *p* = 0.03, and controls, *t*_(30)_ = 7.00, *p* < 0.001. Musicians were significantly more precise than controls on the circle-drawing task, *t*_(28)_ = 3.37, *p* = 0.002. On the finger-tapping task, musicians were significantly more precise than controls, *t*_(28)_ = 2.23, *p* = 0.03, while athletes were not significantly more precise than controls, *t*_(30)_ = 1.87, *p* = 0.07 (Figure 1). The performance of musicians and athletes was not significantly different, *t*_(26)_ = 0.61, *p* = 0.54. We also analyzed the correlation in CV between tasks for each of the groups tested. Results indicated that the variability in the finger-tapping task was not significantly correlated with the variability in the circle-drawing task for any group: musicians (*p* = 0.55), athletes (*p* = 0.08), and controls (*p* = 0.11).

**Figure 2:**
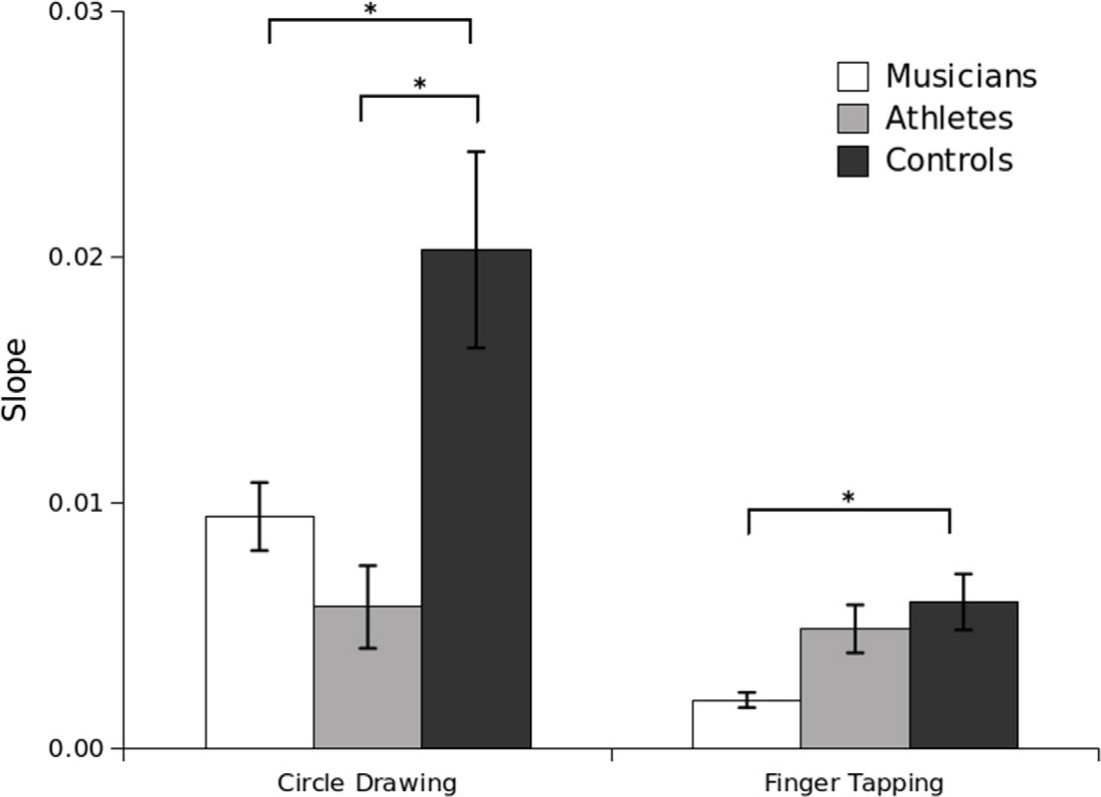
**Slope for the circle-drawing and finger-tapping tasks per group in Experiment 1**. For each participant and for each task, slope values were obtained from a linear regression of detrended variance (averaged across trials) against squared target durations (600 and 800 ms^2^). Lower slope values indicate lower duration-dependent variability. Standard error bars are shown. Significant pairwise differences are marked with an asterisk (^*^p < 0.05).

Slope analysis was next performed to determine whether group differences could be attributed to duration-dependent and/or duration-independent sources. Although the slope analysis was performed with just two target tempi, slope values were almost entirely positive (44 of 46 participants in circle drawing; 45 of 46 participants in finger tapping), indicating greater variance for longer durations (slower tempo), which is consistent with the model's assumptions. An ANOVA on slope values revealed main effects of Task, *F*_(1, 42)_ = 21.01, *p* < 0.001, and Group, *F*_(2, 42)_ = 8.70, *p* < 0.001, as well as a marginal Group × Task interaction, *F*_(2, 42)_ = 2.96, *p* = 0.06. As shown in Figure 2, slope values closely mirrored those for CV. On the circle-drawing task, slope values for athletes (*M* = 0.009) and musicians (0.008) were significantly lower than for controls (*M* = 0.02, *p* = 0.02). However, although the trend was in the same direction, unlike the CV values, athletes' and musicians' slope values did not differ from each other, *p* = 0.88. On the finger-tapping task, slope values were significantly lower for musicians (*M* = 0.002) than for athletes (*M* = 0.004, *p* = 0.03) or controls (*M* = 0.006, *p* = 0.006); as with the CV values, slope values for athletes and controls did not differ from each other (*p* = 0.33). Results of the correlation analysis on the slope values indicated no significant intra-individual correlations for any group. An ANOVA on the intercept values revealed no significant between-subjects effects or interactions. Taken together, the slope analysis indicates that group differences were duration-dependent, suggesting that they can be attributed to the functioning of a timing mechanism rather than to the motor constraints of the tasks.

One generally accepted indicator of the timing strategy adopted in a given task is found through the analysis of lag-one autocorrelation. Tasks that involve an event timing strategy exhibit lag-one autocorrelation values between −0.5 and 0, whereas tasks that involve emergent timing strategies are associated with a non-negative lag-one autocorrelation (Zelaznik and Rosenbaum, 2010; Delignieres and Torres, 2010). Our data were only partially consistent with expectations. One sample *t*-tests [with *p*-value set at 0.01 to control for Type I error (Zelaznik and Rosenbaum, 2010; Baer et al., 2013)] showed that group values were significantly negative in all conditions, which contrasts with the expectation of non-negative lag-one autocorrelations in the (emergent) circle-drawing task. A repeated measures ANOVA revealed that, as expected, lag-one autocorrelation values were significantly more negative in the finger tapping condition (*M* = −0.14) than the circle-drawing condition (*M* = −0.11), *F*_(1, 42)_ = 8.43, *p* < 0.001. Lag-one autocorrelation values, however, did not significantly differ between fast and slow tempo conditions, *F*_(1, 42)_ = 0.19, *p* = 0.66, and the interaction between Task and Tempo also did not reach statistical significance (*p* = 0.24).

Comparing lag-one autocorrelation scores between the different groups, the analysis indicated that there was a significant Group × Task interaction, *F*_(2, 42)_ = 6.81, *p* < 0.001. Examination of the percentage of individuals in each group and condition with significantly negative lag-one correlations, as assessed by one sample *t*-tests on each individual's data, revealed that 60% of athletes and 59% of controls adopted an event-timing strategy to perform the circle-drawing task, whereas 93% of athletes and 88% of the controls used event timing in the finger-tapping task. That is, the percentage of athletes and controls that tended to rely on an event-timing strategy was significantly larger (*p* < 0.001) for the finger-tapping task than for the circle-drawing task. Interestingly, lag-one autocorrelation values for musicians were not significantly different between tasks (*p* = 0.37), and musicians tended to adopt an event-timing strategy to perform both finger-tapping (63%) and circle-drawing tasks (85%).

### Discussion

The results of Experiment 1 demonstrated that movement-based experts were significantly more precise than controls on both timing tasks. Athletes were significantly more precise than controls in the circle-drawing task, and musicians were more precise than controls in the finger-tapping task (Repp, 2005; Repp and Doggett, 2007; Baer et al., 2013). This result suggests that expertise leads to enhanced timing precision in domain-related timing tasks and reinforces a dominant timing skill. This suggestion is supported by results showing that, whereas musicians were significantly more precise than controls in the finger-tapping task, the performance of elite athletes did not differ significantly from controls. This result indicates that the group differences observed in this study can be attributed specifically to the functioning of a timing mechanism rather than motor control in general.

A novel finding of this research is that music training was associated with enhanced precision on a continuous-movement task. Past research has suggested that formal music training only enhances precision of discrete movements but not continuous movements (Baer et al., 2013). It should be acknowledged that the use of a computer mouse to perform the tasks might have influenced the results. However, task constraints cannot readily account for the discrepancy between our results and those reported by Baer et al. (2013). The slope analysis suggests that our results are best explained by the functioning of a timing mechanism rather than by the constraints of the tasks. Hence, it can be speculated that group differences may contribute to the discrepancy of results in these studies, such as number of years of formal music training, instrument of expertise, amount of current involvement in musical activities, or age of commencement of training. Research is needed to assess the extent to which these factors contribute to the development of timing skills.

The finding that music training was associated with enhanced precision on the continuous-movement task is compatible with the hypothesis that the distinction between event and emergent timing is not as rigid as initially proposed, and that these mechanisms are not strictly tied to specific tasks such as tapping and circle drawing (Jantzen et al., 2002, 2004; Repp and Steinman, 2010; Studenka et al., 2012; Studenka, 2014). The hypothesis that the dissociation between event and emergent timing is not an all-or-nothing process (Repp and Steinman, 2010; Studenka et al., 2012) implies that the circumstances in which the different timing modes are employed are open for investigation. In Experiment 1, lag-one autocorrelation values were significantly negative in all conditions, suggesting that participants tended to adopt an event-timing strategy for both discrete and continuous tasks. More specifically, approximately 60% of participants in each group tended to adopt event-timing strategies to perform the circle-drawing task. Interestingly, whereas the percentage of athletes and controls that adopted event timing was higher for finger tapping than for circle drawing, the percentage of musicians that relied on event timing was not statistically different between tasks. One interpretation of this result is that years of formal music training prompted participants to rely on event-timing mechanisms to perform any timed movement, even when those movements are continuous (Studenka et al., 2012; Baer et al., 2013).

In Experiment 2, we further explored the hypothesis that movement-based expertise is associated with enhanced skill in discrete and continuous movement, while reinforcing one predominant timing mode. We also reexamined recent evidence that when participants are engaged in a timing task, the presence of salient feedback that defines the completion of cyclical time intervals elicits timing behavior consistent with event timing, even for continuous-movement tasks (Zelaznik and Rosenbaum, 2010; Studenka et al., 2012). Studenka et al. (2012) showed that the introduction of discrete tactile events presented at the completion of each cycle of movement induced event timing in a typically emergent timing task. This finding corroborated a previous study that suggested that event timing can be elicited by the insertion of regular cycles of auditory feedback (Zelaznik and Rosenbaum, 2010). To examine these issues, in Experiment 2 we tested whether the presence of auditory feedback elicits an event-timing strategy for a circle-drawing task among participants with intense musical or athletic training.

## Experiment 2

### Materials and methods

#### Participants

Thirty-one elite athletes (10 females) were recruited from Macquarie University through the Elite Athlete Scholarship Program. Athletes' average age was 21.06 years old (*SD* = 3.69, range 18–32 years) and they had been involved in athletic training for an average of 8.31 years (*SD* = 5.55). Athletic training included sports that require discrete interactions with a ball or other projectile (e.g., kicking, catching, or repelling a ball in soccer, rugby, or volleyball) and sports that primarily involve continuous movements (e.g., strokes in swimming, cycling, rowing). None of the athletes were involved in the first experiment and none had had more than 2 years of musical training. Musicians (*n* = 17, 15 females) were recruited through the Departments of Music and Psychology at Macquarie University and local conservatories and universities. The average age of musicians was 20.72 years (*SD* = 3.52, range 18–29 years). Musicians were all currently involved in music activities for a minimum of 2 h/week and all had been involved in formal music training for at least 10 consecutive years (*M* = 11.94, *SD* = 2.68). None of the participants were involved in the previous study. Musicians played a range of instruments, including piano, guitar, and violin. Control participants (*n* = 10, 10 males) were postgraduate or professional computer programmers recruited through the Computer Science Department at Macquarie University. Participants were on average 31.58 years old (*SD* = 7.21, range 22–49 years), and had an average of 10 years of training in their area of expertise (*SD* = 6.07), and reported that they had no previous formal athletic training and no significant past or current involvement in music. Because the control group consisted of professionals and postgraduate students, there was a significant group difference in mean age [*F*_(2, 55)_ = 26.09, *p* < 0.001]. All participants provided informed consent and were debriefed about the goals of the experiment. Fifty-six participants were right-handed and two were left-handed, and all participants reported that they had no hearing or motor impairment. Participants received financial compensation for their participation.

#### Materials, stimuli, procedure, and data analysis

Stimulus presentation and data collection involved the same equipment as in Experiment 1, with the exception that participants completed the tasks using the laptop's touch pad in order to facilitate performance on the circle-drawing task. Procedures and data analysis followed the protocol established in Experiment 1. The main change was the introduction of auditory feedback at the continuation phase of the task. For each trial, after participants synchronized their movements (circle drawing or finger tapping) with an isochronous metronome for 18 pacing signals, the metronome stopped and participants were instructed to continue to produce 36 more movements at the tempo established by the metronome.

For the finger-tapping task, participants repeatedly tapped on the touch pad with their right index finger at the tempo set by the metronome. In the continuation phase, every tap triggered a feedback tone of 40 ms duration with a fundamental frequency of 480 Hz and at an intensity of 74 dB SPL. In the circle-drawing task, participants repeatedly traced an unfilled circle template of 5 cm in diameter displayed on the screen with the mouse cursor using their right index finger in time with the metronome and continued the task in the absence of the external timing cue. Participants were told to pass the cursor over a crossing intersection at 270° of the circle in synchrony with the metronome. In the continuation phase, every time the cursor trajectory crossed the intersection the auditory feedback was provided.

### Results

Participants were accurate in maintaining the target tempo during the continuation phases of trials [fast tempo (600 ms IOI): *M* = 613 (*SD* = 25); slow tempo (800 ms IOI): *M* = 791 (*SD* = 39)]. An analysis of mean IRI across the two tasks showed no significant group differences or group interactions. That is, all three groups maintained a similar overall tempo in the continuation phase of the timing tasks.

To measure timing precision, CV scores were averaged by task and tempo for each participant and entered into a mixed design ANOVA with Task (circle drawing, finger tapping) and Tempo (slow, fast) as within factors and Group (athletes, musicians, controls) as the between-subjects factor. The analysis revealed a significant main effect of Task, *F*_(1, 55)_ = 4.60, *p* = 0.03, and a paired sample *t*-test confirmed that across the three groups performance on the finger-tapping task (*M* = 0.05) was significantly more precise than on the circle-drawing task (*M* = 0.10), *t*_(57)_ = 6.87, *p* < 0.001. There was also a main effect of Tempo, *F*_(1, 55)_ = 35.61, *p* < 0.001, and a significant interaction between Task and Tempo, *F* = 17.69, *p* < 0.001. Results indicated that precision was significantly better for fast tempo (*M* = 0.05, *p* < 0.001) than slow tempo (*M* = 0.11) in the finger-tapping task. Participants were also significantly more precise in fast tempo (*M* = 0.06) than slow tempo (*M* = 0.09, *p* < 0.001) in the circle-drawing task.

**Figure 3:**
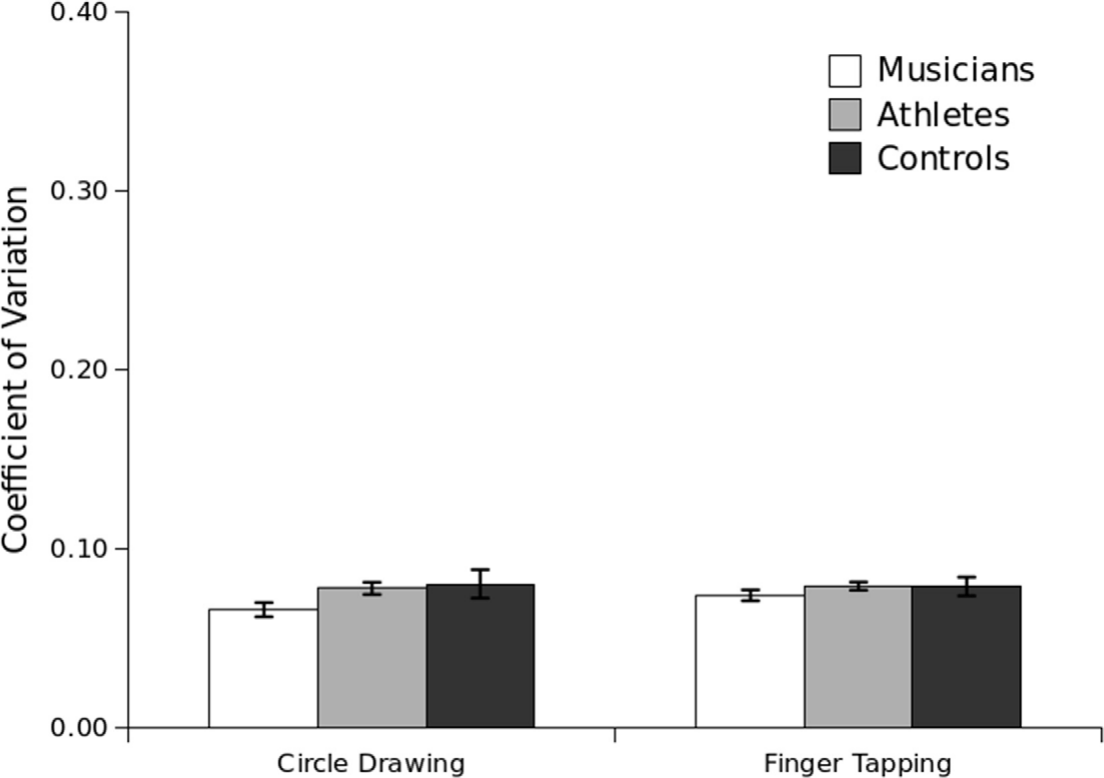
**Coefficient of Variation (CV) per group in Experiment 2**. Standard error bars are shown.

Between-subjects analysis indicated that there was a significant main effect of Group, *F*_(2, 55)_ = 3.23, *p* = 0.04, and a marginally statistical interaction between Task, Tempo and Group, *F*_(2, 55)_ = 2.81, *p* = 0.06. Analysis of the circle-drawing task showed that musicians were significantly more precise than controls on the circle-drawing task (*p* = 0.01), but there was no statistical difference between the performance of athletes and musicians (*p* = 0.24), or between athletes and controls (*p* = 0.07). A similar pattern was observed for the finger-tapping task, which also corroborated the results of Experiment 1: musicians were significantly more precise than controls (*p* = 0.04), but no other significant group differences were observed (athletes and controls, *p* = 0.14; musicians and athletes, *p* = 0.33; see Figure 3).

Different subgroups of athletes were included in the study (e.g., swimming, rowing, rugby, volleyball, squash, triathlon, ice hockey, martial arts, and others). We also examined whether performance differed between athletes specializing in sports that require discrete interactions with a ball or other projectile (e.g., kicking, catching, or repelling a ball in soccer, rugby, or volleyball) and athletes trained in continuous movements (e.g., strokes in swimming, cycling, rowing). An independent sample *t*-test indicated that there was no statistical difference between athletes of sports based on different movement class on either the circle-drawing task, *t*_(29)_ = 1.40, *p* = 0.17, or the finger-tapping task, *t*_(29)_ = 0.31, *p* = 0.75.

**Figure 4:**
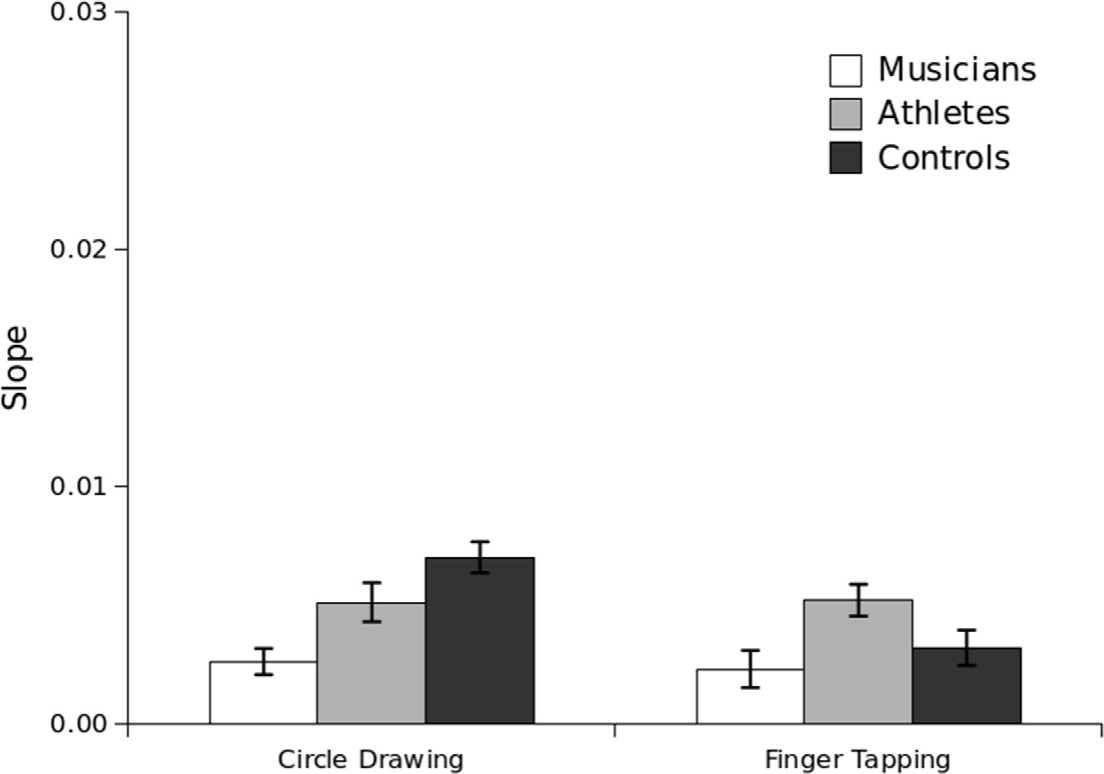
**Slope for the circle-drawing and finger-tapping tasks per group in Experiment 2**. Standard error bars are shown.

Slope analysis was next conducted to determine whether, as in Experiment 1, the group differences in CV could be isolated to duration-dependent variability. As shown in Figure 4, a close correspondence was again observed. As with the CV values, only a main effect of Group was significant, *F*_(2, 55)_ = 3.79, *p* = 0.03. Slope values, like CV values, were lower for musicians (*M* = 0.002) than athletes (*M* = 0.005; *p* = 0.03) and controls (*M* = 0.005; *p* = 0.02), but did not differ between athletes and controls (*p* = 0.96). In contrast, an ANOVA on the intercept values revealed no significant between-subjects effects or interactions, and intraindividual correlations on the slope values were also not significant for any group. Thus, as in Experiment 1, group differences in total variability (as indexed by CV) could be isolated to duration-dependent variability (e.g., arising from noise in a central timekeeping mechanism) rather than duration-independent differences associated with the motor implementation of these tasks.

**Figure 5:**
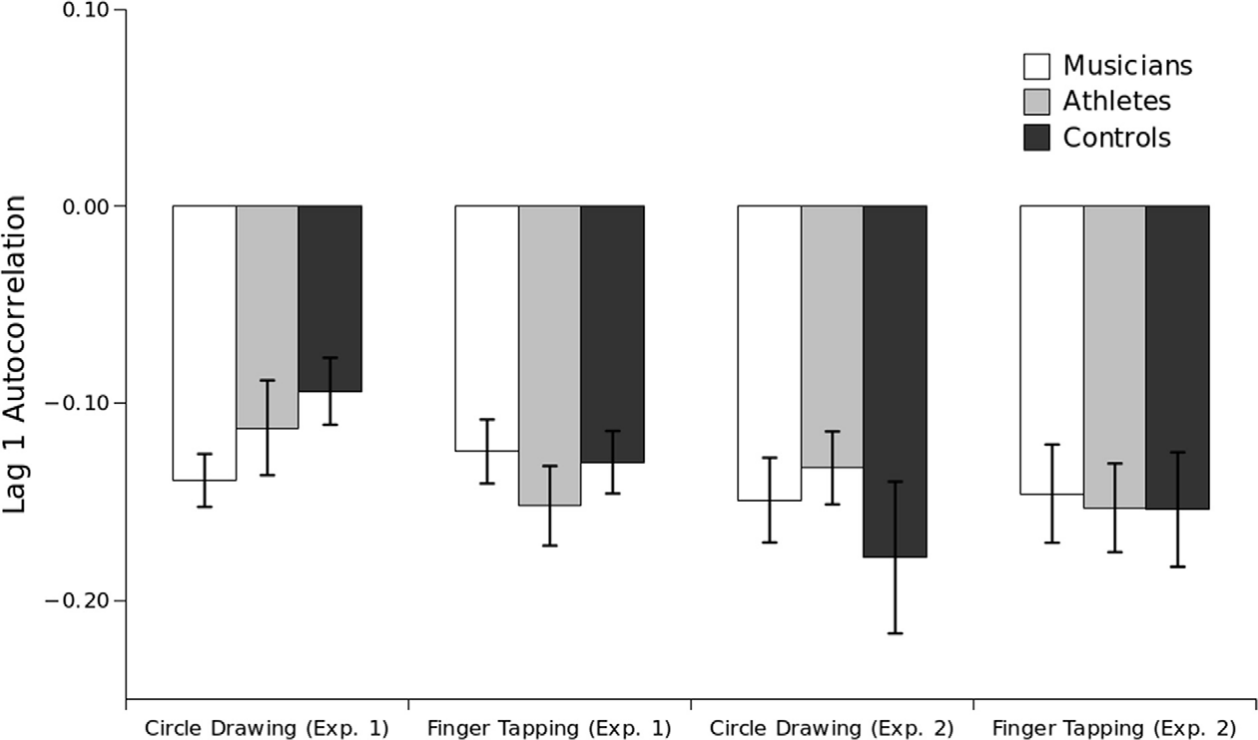
**Lag-one autocorrelation values averaged across tempi by Group and Experiment on the circle-drawing and finger-tapping tasks**. Auditory feedback was provided in Experiment 2 only. Note: Groups of participants in Experiment 2 are different from those in Experiment 1.

Previous research has suggested that the introduction of a perceptual event, such as tactile or auditory feedback, can strongly induce event-timing strategies (as indexed by negative lag-one autocorrelations) even for tasks performed with continuous, smoothly-produced movements (Zelaznik and Rosenbaum, 2010; Studenka et al., 2012). Our data were generally consistent with these findings. One sample *t*-tests showed that group means were significantly negative in all conditions (see Figure 5). A mixed-design ANOVA with Task (circle drawing, finger tapping) and Tempo (slow, fast) as within-subject factors, and Group (athletes, musicians, controls) as the between-subjects factor, revealed that lag-one autocorrelations values were not significantly different between tasks [*F*_(1, 55)_ = 0.21, *p* = 0.64]. Lag-one autocorrelation values were significantly different between fast and slow conditions in Experiment 2, *F*_(1, 55)_ = 6.23, *p* = 0.01, and there was also a significant interaction between Task and Tempo (*p* = 0.002). Pairwise comparisons indicated that there was a significant difference between lag-one autocorrelation scores in the slow (*M* = −0.11) and fast conditions (*M* = −0.17) for the finger-tapping task (*p* = 0.001), but lag-one autocorrelation values did not significantly differ between the slow (*M* = −0.13) and fast conditions (*M* = −0.12) for the circle-drawing task (*p* = 0.70).

An examination of the percentage of individuals in each group and condition with significantly negative lag-one autocorrelations, as assessed by one sample *t*-tests on each individual's data, revealed that 90% of participants in the control group adopted an event-timing strategy to perform the circle-drawing task in Experiment 2, and 60% of participants in this group used event timing to perform the finger-tapping task. Among movement-based experts, the percentage of individuals that adopted an event-timing strategy to perform the circle-drawing and finger-tapping tasks was similar for musicians (76%) and athletes (68%). ANOVA confirmed that there was no interaction between Group (musicians, athletes, controls) and Task (finger tapping, circle drawing), *F*_(2, 55)_ = 0.98, *p* = 0.38. Taken together, these results suggest that the majority of participants adopted an event-timing strategy to perform both tasks in Experiment 2 (Table 1).

**Table 1:** **Percentage of individuals with significantly negative lag-one autocorrelation values for each group and condition in Experiment 1 (no auditory feedback) and Experiment 2 (with auditory feedback), and Event Timing Index (ETI: the percentage of individuals with negative lag-one autocorrelation/meanCV)**.

**Measures of event timing**		
	**Experiment 1 Circle drawing Percentage (ETI)**	**Experiment 2 Circle drawing Percentage (ETI)**
Musicians	85% (8.3)	76% (8.1)
Athletes	60% (5.8)	68% (6.8)
Controls	59% (3.3)	90% (8.6)
All	67% (5.2)	74% (7.5)
	**Finger tapping**	**Finger tapping**
Musicians	62% (13.1)	76% (16.6)
Athletes	93% (14.0)	68% (12.5)
Controls	88% (11.8)	60% (10.1)
All	82% (13.1)	70% (13.0)

To test whether the presence of auditory feedback defining the completion of cyclical time intervals influenced the timing strategy adopted, we examined the percentage of individuals in each group and condition that had significantly negative lag-one autocorrelations between Experiments 1 and 2 (see Table 1). Examination of Table 1 suggests that the use of event timing depended on the condition and expertise of the participant. First, for the circle-drawing task, a smaller percentage of control (non-expert) participants used an event-timing strategy when there was no auditory feedback (59%) than when there was auditory feedback (90%). Second, when there was no auditory feedback, musicians were more likely to use an event-timing strategy (85%) than control participants (59%).

However, interpreting the raw percentage of negative autocorrelation values is complicated by the fact that these values not only reflect a tendency to adopt an event timing strategy; they also reflect timing variability (van Beers et al., 2013). Thus, changes in the percentage of negative (lag one) autocorrelation values may reflect a change in the strategies adopted by participants; a change in the average variability of timing; or both. For this reason, it is useful to consider the percentage of negative autocorrelation values *relative* to the average CV of participants in each condition. Thus, we defined the *Event Timing Index* (ETI) as the percentage of participants with negative lag-one autocorrelations divided by the CV averaged across these same participants. This normalized measure of event timing permits a meaningful comparison between conditions.

Table 1 displays ETI values in parentheses. These values suggest that across groups and experiments, an event-timing strategy was used significantly less for circle drawing (mean ETI = 6.6) than for finger tapping (mean ETI = 13.0). For circle drawing, there was a greater tendency for control participants to adopt an event timing strategy when there was auditory feedback (ETI = 8.6) than when there was no auditory feedback (ETI = 3.3). This finding suggests that, for circle drawing, the presence of a salient perceptual event defining the completion of cyclical time intervals influenced the timing strategy adopted by non-experts.

Musicians, however, exhibited a comparatively strong tendency to employ event timing when performing the circle-drawing task regardless of whether there was or was not auditory feedback (ETI = 8.3 and 8.1, respectively). For finger tapping, the tendency to adopt an event-timing strategy was similar in the two Experiments (mean ETI in Exp 1 = 13.1; mean ETI in Exp 2 = 13.0). In short, introducing a salient perceptual event demarcating the completion of each movement cycle encouraged an event-timing strategy for circle drawing, but not finger tapping. One interpretation of this finding is that there was already a strong tendency to employ an event-timing strategy for finger tapping, so the inclusion of auditory feedback had no additional impact on this tendency.

### Discussion

The findings of Experiment 2 confirmed that participants performed significantly more precisely in the finger-tapping task than in the circle-drawing task. The results also indicated that precision was significantly better for the fast-tempo condition than the slow-tempo condition in both finger-tapping and circle-drawing tasks. Previous studies have also reported significant interactions between task precision and tempo, suggesting that the timing mechanism adopted is affected by the rate of timed movements (Huys et al., 2008; Repp, 2008; Zelaznik and Rosenbaum, 2010). The slope analysis also suggests that the differences in total variability cannot be attributed to differences associated with the motor implementation of the tasks, but to duration-dependent variability (e.g., in event timing, a central clock mechanism accumulates error as the interval duration increases).

The results of Experiment 2 also confirmed that musicians were significantly more precise than controls at both finger tapping and circle drawing. It can be suggested that music training may engage and refine both discrete and continuous movements. One explanation for this result is that both event and emergent timing are implicated in the accurate timing achieved by elite performers, and that music training leads to the enhanced operation of both timing modes (Jantzen et al., 2002, 2004; Repp and Steinman, 2010; Studenka et al., 2012; Studenka, 2014). However, it is also possible that the superior performance by musicians on both discrete and continuous tasks could be largely attributable to an enhanced central clock mechanism, as the results of the lag-one autocorrelation analysis suggested that the vast majority of musicians tended to employ an event-timing strategy to perform both discrete and continuous tasks.

In Experiment 2, the performance of athletes in the circle-drawing task did not differ significantly from that of controls, in contrast to the results of Experiment 1. This discrepancy could be related to the fact that 90% of controls and 68% of athletes adopted an event-timing strategy to perform the continuous-movement task when auditory feedback was available (Experiment 2), whereas 59% of controls and 60% of athletes used event timing to perform the circle-drawing task when auditory feedback was not available (Experiment 1). In other words, in the presence of auditory feedback there was a greater tendency to adopt an event timing strategy to perform the circle-drawing task, especially for participants without movement-based expertise. This finding corroborates previous evidence that the introduction of a perceptual event, such as tactile or auditory feedback, induces an event-timing strategy even for tasks performed with continuous movements (Zelaznik and Rosenbaum, 2010; Studenka et al., 2012). This tendency may explain why the precision of continuous movements increases when auditory feedback is available (Zelaznik and Rosenbaum, 2010). More generally, it is known that the presence of feedback significantly enhances timing precision Aschersleben et al., 2001; Aschersleben, 2002; Stenneken et al., 2006; Goebl and Palmer, 2009, and event timing is preferred in synchronization tasks, given that discrete actions are less variable and quicker to adjust after perturbations in the sensory input (Elliot et al., 2009).

Taken together, the results of Experiment 2 corroborate findings obtained in Experiment 1 that movement-based expertise significantly improves timing skills, and that extensive training in music leads to enhanced precision for both discrete and continuous movements. The findings also support the hypothesis that event and emergent timing are not uniquely tied to specific types of movements but can be influenced by expertise (Jantzen et al., 2002, 2004; Zelaznik and Rosenbaum, 2010), the presence of feedback (Studenka and Zelaznik, 2011), and movement speed (Huys et al., 2008).

## General discussion

This investigation sought to examine the effects of expertise and training on the precision of timed movements. The results are compatible with the view that movement-based training significantly enhances the precision of timing skills, and that this effect depends on the nature of the training. It was also observed that expertise is an important predictor of the timing mechanism that is engaged during timed actions. These findings help to clarify the distinction between event and emergent timing mechanisms by showing that expertise and training can influence the timing mode that is employed in a particular movement-based task.

Experiment 1 demonstrated that athletes were significantly more precise in the production of continuous rhythmic movements, whereas musicians were significantly more precise in discrete rhythmic movements in the absence of auditory feedback. These results indicate that intense training and expertise can help to improve timing precision, and corroborate the initial hypothesis that music performance relies predominantly on event timing (Repp and Doggett, 2007; Baer et al., 2013; Albrecht et al., 2014), whereas athletic activities tend to employ more predominantly smooth and continuous movements based on emergent timing (Kelso et al., 1981; Sternad et al., 2000; Jaitner et al., 2001; Jantzen et al., 2008; Balague et al., 2013). Thus, hours of daily practice involving a predominant type of movement (i.e., discrete or continuous) may reinforce one dominant timing mode. This finding is particularly relevant to the development of educational and rehabilitation programs that could greatly benefit from activities targeting specific classes of movements.

It is important to state, however, that actions can be implemented in different ways (e.g., walking vs. marching) and may often engage multiple mechanisms simultaneously. For example, playing the piano not only requires precise timing of the pianist' keystrokes but also a fluid transition of the hand across the piano keys. Rowing or swinging a badminton racquet, on the other hand, are continuous actions in the sense that the movement is not smooth and interrupted; however they are discrete insofar as movements are segmented by perceptual events (e.g., contact of the oar with the water, and the racquet with the shuttlecock). Therefore, whereas it is possible to isolate discrete and continuous movements in laboratory for experimental purposes, performances often require both classes of rhythmic actions (Sternad et al., 2000; Hogan and Sternad, 2007; Sternad, 2008; Repp and Steinman, 2010; Studenka, 2014). The results of the present study do not support the idea that musical and athletic skill are associated with event-timing and emergent timing, respectively. On the contrary, our findings suggest that to accurately perform timing tasks at high skill level, experts may rely on both timing modes, although one timing mechanism is often dominant. Therefore, an essential skill in movement-based expertise is to smoothly transition between movements of different classes.

Our findings are consistent with the idea that event and emergent timing mechanisms are not strictly tied to specific tasks (Jantzen et al., 2002, 2004; Repp and Steinman, 2010; Studenka et al., 2012; Studenka, 2014). First, musicians were not only significantly more precise than controls in the finger-tapping task but also in the circle-drawing task, suggesting that music training refines both discrete and continuous rhythmic movements. Second, lag-one autocorrelation values were significantly negative in all conditions in Experiment 1, suggesting that participants tended to adopt an event-timing strategy to perform both discrete and continuous tasks, even when no salient perceptual event was present.

The analysis of the Event Timing Index (ETI) allowed us to further investigate the effect of auditory feedback on the timing strategy adopted to perform continuous movements. These results suggested that the percentage of musicians that used an event-timing strategy to complete the circle-drawing task did not change significantly when auditory feedback was provided at the end of each movement cycle. Years of formal music training might have prompted participants to rely on event-timing mechanisms to complete a continuous-movement task (Studenka et al., 2012; Baer et al., 2013; van Beers et al., 2013). These findings support the suggestion that expertise and training are important predictors of the timing mechanism engaged in maintaining precise timed actions. On the other hand, the percentage of participants who adopted event timing to complete the circle-drawing task significantly increased when auditory feedback was present, especially for control participants. For this task, 59% of participants tended to use an event-timing strategy when no auditory feedback was provided (Experiment 1), but 90% of the control group adopted an event-timing strategy when auditory feedback was provided (Experiment 2). This finding indicates that salient events (e.g., auditory, tactile) signaling the completion of a movement cycle can be used to generate an internal representation of the time intervals to be produced based on clock-like mechanisms (Zelaznik and Rosenbaum, 2010; Studenka et al., 2012). It is known that sensory feedback enhances timing accuracy (Aschersleben et al., 2001; Rabin and Gordon, 2004; Repp, 2005; Goebl and Palmer, 2009; Gray, 2009). However, it is important to note that the manipulation of auditory feedback is possible in experimental conditions, but in real life circumstances multiple sources of feedback may be used to monitor and refine the accuracy and precision of timed actions (Aschersleben et al., 2001). Future studies are needed to examine the role of event and emergent timing mechanisms in the control of discrete and continuous rhythmic movements in ecologically valid conditions. Such research would shed light on the relative importance of these two timing strategies for the production of accurately timed movements in real-life circumstances.

It should be acknowledged that certain methodological features of our investigation limit the conclusions that can be drawn. First, we observed that lag-one autocorrelation values were significantly negative in all conditions in both experiments. This finding suggests that participants tended to adopt an event-timing strategy to perform both discrete and continuous tasks, even when no salient perceptual event was present. One explanation for this finding is that participants took part in the tapping task before the circle-drawing task, as in previous studies (Zelaznik and Rosenbaum, 2010; Studenka et al., 2012), and task order may have significantly influenced the timing strategy adopted by some participants. Previous research has suggested that practicing one timing task reinforces a particular timing strategy, which may then persist over time and over tasks (Jantzen et al., 2002, 2004; Studenka et al., 2012). Interestingly, this carry-over effect may have been stronger for some participants than others. Nonetheless, this possibility also corroborates a central conclusion of the study: that timing strategies are not strictly tied to specific tasks but may be influenced by factors such as task order, expertise and training, and the presence of salient perceptual events.

Second, it is important to acknowledge that different groups of participants were used in the two experiments, preventing a within-subject comparison between the results of these experiments. For this reason, it is difficult to estimate that the precise effect of auditory feedback on the timing strategy adopted. To overcome this limitation, we developed the “Event Timing Index (ETI).” This index is essentially the relationship between the percentage of participants with negative lag-one autocorrelations divided by CV averaged across these same participants. The results of this analysis strongly suggest that auditory feedback influenced the timing strategy adopted for circle drawing but not finger tapping. Further research is required to validate this conclusion.

In summary, expertise in sports and music is significantly associated with enhanced precision of timing skills, but this effect depends on the nature of the expertise and the presence of auditory feedback. It should be emphasized that one interpretation of these findings is that individuals with superior timing precision gravitated to these pursuits. However, it is likely that expertise and training further helped to engage and refine mechanisms associated with skilled timing. Expertise was also an important predictor of the type of timing mechanism that individuals employed for both discrete and continuous movements, which casts further doubt on the long-standing assumption that event and emergent timing mechanisms are strictly tied to discrete and continuous movement tasks, respectively.

## Author contributions

Thenille Braun Janzen was the major contributor to this co-authored paper and took primary responsibility for recruiting participants, conducting the experiment, analysing and interpreting the data and preparing the manuscript. William Forde Thompson, Paolo Ammirante, and Ronald Ranvaud provided input on one or more of the experimental design, data analysis and interpretation, and manuscript preparation.

### Conflict of interest statement

The authors declare that the research was conducted in the absence of any commercial or financial relationships that could be construed as a potential conflict of interest.
